# Soft X-ray spectromicroscopy of human fibroblasts with impaired sialin function†

**DOI:** 10.1039/d4ra05520a

**Published:** 2024-09-10

**Authors:** Tuomas Mansikkala, Salla M. Kangas, Ilkka Miinalainen, Pia Angervaniva, Niklas Darin, Maria Blomqvist, Reetta Hinttala, Marko Huttula, Johanna Uusimaa, Minna Patanen

**Affiliations:** a Nano and Molecular Systems Research Unit, 90014 University of Oulu PO Box 3000 Finland leo.mansikkala@oulu.fi minna.patanen@oulu.fi; b Biocenter Oulu, 90014 University of Oulu PO Box 5000 Finland; c Research Unit of Clinical Medicine and Medical Research Center, Oulu University Hospital and University of Oulu 90014 Oulu Finland; d Department of Pediatrics, Institute of Clinical Sciences, Sahlgrenska Academy, University of Gothenburg 40530 Gothenburg Sweden; e Department of Laboratory Medicine, Institute of Biomedicine, University of Gothenburg, Sweden and Department of Clinical Chemistry, Sahlgrenska University Hospital 41345 Gothenburg Sweden; f Department of Paediatrics and Adolescent Medicine, Paediatric Neurology Unit, Oulu University Hospital 90029 Oulu Finland

## Abstract

Salla disease (SD) is a lysosomal storage disease where free sialic acid (SA) accumulates in lysosomes due to the impaired function of a membrane protein, sialin. Synchrotron radiation-based scanning transmission soft X-ray spectromicroscopy (STXM) was used to analyze both SD patients' fibroblasts and normal human dermal fibroblasts (NHDF) from healthy controls. Both cell lines were also cultured with *N*-acetyl-d-mannosamine monohydrate (ManNAc) to see if it increased SA concentration in the cells. The STXM technique was chosen to simultaneously observe the morphological and chemical changes in cells. It was observed that free SA did not remain in the lysosomes during the sample processing, leaving empty vacuoles to the fibroblasts. The total cytosol and entire cell spectra, however, showed systematic differences between the SD and NHDF samples, indicating changes in the relative macromolecular concentrations of the cells. The NHDF cell lines contained a higher relative protein concentration compared to the SD cell lines, and the addition of ManNAc increased the relative protein concentration in both cell lines. In this study, two sample preparation methods were compared, resin-embedded thin sections and cells grown directly on sample analysis grids. While the samples grown on the grids exhibited clean, well-resolved spectra not masked by embedding resin, the low penetration depth of soft X-rays hindered the analysis to only the thin region of the microfilaments away from the thick nucleus.

## Abstract 2

A comparative study of normal human fibroblasts and Salla disease patients' fibroblasts reveals changes in the soft X-ray spectroscopic signatures of disease cells. Synchrotron radiation imaging was performed for thin sections and grid-grown cells.

## Graphical abstract

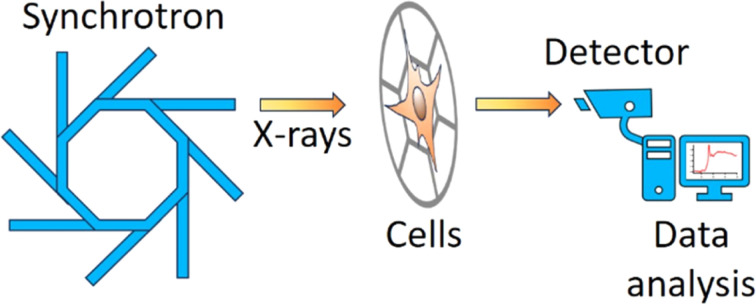

## 1. Introduction

One of the significant challenges in the imaging of biomaterials is to obtain simultaneously high spatial resolution images while preserving the ultrastructural features and chemistry of the materials as intact as possible. While electron microscopy (EM) and super-resolution fluorescence nanoscopy are extremely powerful techniques to which a large body of biomedical imaging work relies on, techniques that do not require staining or fluorescent labelling are under constant development. In tissue imaging, spectromicroscopic techniques that utilize the intrinsic chemical contrast of biological tissue have the benefit of being applicable to cases where selective labeling agents are not available. They also avoid the structural and chemical changes that staining can introduce. Synchrotron radiation (SR) facilities offer electromagnetic radiation from infrared wavelengths to hard X-rays, thus offering various possibilities for label-free spectromicroscopic tissue imaging. Fourier transform infrared micro-spectroscopy (FTIRM) performed at synchrotrons benefit from the much higher brightness of the source compared to lab-based thermal sources, thus improving the spectral quality significantly for smaller illumination areas.^1^ The chemical contrast is obtained due to the molecule-specific vibrational modes, allowing the differentiation of macromolecules and also their structure, *e.g.*, the misfolding and aggregation of proteins.^2^ The energies of the vibrational modes of molecules are dependent on chemical bonds, as are the excitation energies of electrons in the molecules. X-ray absorption near edge structure (XANES) spectroscopy uses this selectivity of excitation energy for chemical characterization. Biological tissues consist mostly of the light elements H, C, N, and O; their electronic excitation and ionization energies match with ultraviolet and soft X-ray radiation. For example, in order to make an excitation in which the C 1s electron is promoted to an unoccupied orbital in a C atom part of a phenyl group (C 1s → π* transition), a 285 eV energy photon is needed.^3^ If the excitation takes place in a C atom part of a carboxylic group, a somewhat higher energy is needed, typically around 288 eV.^3^ Different cell organelles and tissue components vary in their relative amount of macromolecules with specific functional groups, and by recording the C 1s XANES spectra pixel-by-pixel by scanning the X-ray beam across the studied sample, it is possible to form a map of the tissue chemistry. This is the principle of scanning transmission X-ray microscopy combined with XANES (STXM-XANES).^4,5^ The SR beam can be focused with Fresnel zone plates down to a 10 nm spot, offering much higher spatial resolution than FTIRM. However, due to low penetration depth or in turn the high absorption cross-section of soft X-rays, the tissue to be imaged at C, N, and O edges needs to be cut in to thin sections, and thus the technique is in that sense more limited. Hard X-rays, in contrast, can easily penetrate soft tissue and are thus daily used in the imaging of macroscopic objects, like humans, in computed tomography. Heavier elements, like Fe, have their deep core-excitation energies in the hard X-ray energy range, and can be used for XANES imaging.^6^ At SR facilities, the extreme coherence of the hard X-rays offers a possibility for ptychographic nanotomography, allowing 3D imaging of biological specimens close to their native state with sub-micron resolution.^7^ Also, the combination of synchrotron-based FTIRM and STXM techniques has been recently used; for example, for cell^8^ and protein-based biomaterial studies.^9^

STXM-XANES is actively used to study deposits in human tissues, especially related to neurodegenerative diseases, like Alzheimer's disease.^10–14^ Cosmidis *et al.* localized apatite precipitates in a bone-like collagen matrix using correlative imaging at the C 1s and Ca 2p edges.^15^ STXM-XANES has also been used to study the penetration of topically applied drugs into the skin.^16–18^ Shinohara *et al.* studied the distribution of DNA, RNA, histone, and proteins in cells, which were directly applied to X-ray transmission grids and membranes without embedding.^19–21^ In our recent work, we used C and O 1s edge STXM-XANES to study resin-embedded thin sections of the liver and kidney tissues of mice.^22^

In this work, the STXM-XANES technique was used to study cultured fibroblasts of Salla disease (OMIM # 604369) patients and control cell lines. Salla disease (SD) is caused by a variant in the *SLC17A5* gene that encodes sialin, a lysosomal membrane protein that transports sialic acid (SA) out of lysosomes.^23^ A high-resolution structure of sialin was just recently published based in a cryo-EM study.^24^ The impaired function of sialin causes free SA to accumulate, and SD patients can have approximately 10–100 times the normal amounts of free SA in their tissues.^25,26^ Electron microscopy of patient-derived fibroblast has shown that the cytoplasm has clear vacuolar structures, the minority of which have an amorphous weakly OsO_4_-stained material.^25^*De Novo* sialic acid biosynthesis occurs in the cytoplasm. In the sialic acid synthesis pathway, *N*-acetylmannosamine (ManNAc) is a precursor to sialic acid.^27^ Early studies done in fibroblasts indicated that ManNAc supplementation to the cultured cells leads to increased sialic acid biosynthesis, and the excessive accumulation of sialic acid into lysosomes was observed in fibroblasts obtained from SD patients.^28^

The aim of the present study was to investigate SA accumulation in SD patient fibroblasts compared to healthy control fibroblasts based on the native X-ray absorption contrast at C 1s edge energies. The effects of ManNAc exposure to the cell morphology and chemistry in patient and control cells were also investigated. For sample preparation, two approaches were used: one using thin sections of fibroblasts and another growing fibroblasts directly on electron microscopy grids. Cells grown directly on grids have the benefit of avoiding resin embedding, which supports the structures during the sectioning.

## 2. Materials and methods

### 2.1 Cell culture

Two human cell lines were used for the experiments: the human dermal fibroblast cell line derived from SD patients harboring the homozygous *SLC17A5* variant p.Arg39Cys (c.115C > T, NM_012434.5) (from Biobank Väst (Gothenburg, Sweden) and the commercially available healthy control normal human dermal fibroblasts cell line (NHDF, PromoCell, Heidelberg, Germany). The research plan, including the studies on patient-derived fibroblasts, was approved by the Ethics Committee of the Northern Ostrobothnia Hospital District, Oulu University Hospital, Oulu, Finland (EETTMK: 68/2017, GENOLED), including an amendment accepted on the 19th of April 2021).

**Fig. 1:**
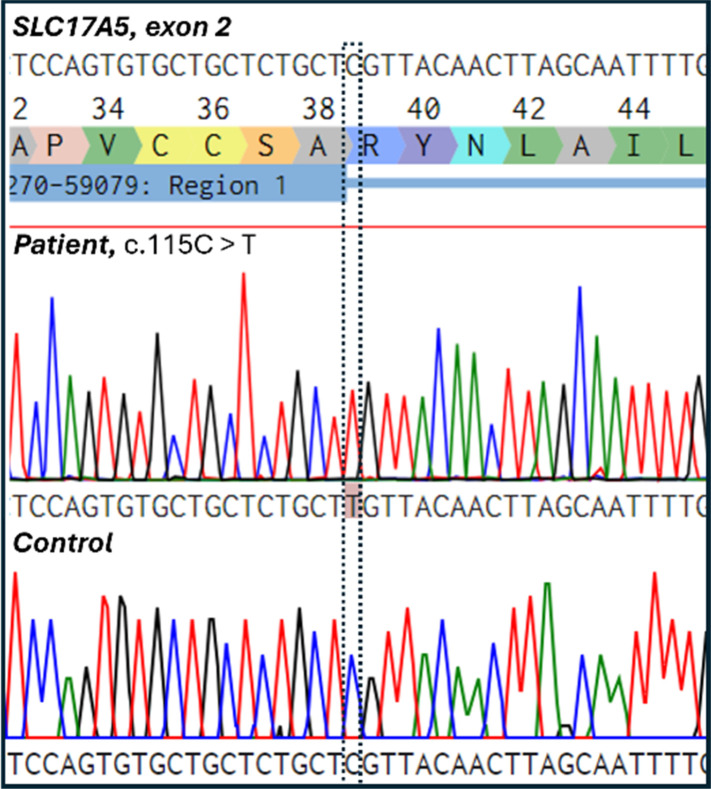
Electropherogram showing the Salla disease-causing homozygous nucleotide change c.115C > T (p.Arg39Cys) in the patient cell line. The control cell line did not contain the variant.

It was confirmed that the *SLC17A5* c.115C > T (p.Arg39Cys) variant was located in exon 2 by Sanger sequencing of DNA from the patient-derived cell line (Fig. 1). Sanger sequencing was carried out at the Biocenter Oulu Sequencing Center (University of Oulu, Finland) using the following primers flanking the variant: the forward primer 5′-ACCTGCTTTTCTCATGCCGT-3′ that binds to the intronic region before exon 2 and the reverse primer 5′-GGACACGCCTTGGAAGTTCT-3′ that binds to the end of the exon 2. The sequence shows a missense variant where nucleobase cytosine (C) is replaced by thymine (T) leading to the amino acid substitution of arginine (R) to cysteine (C). This variant has been shown to be prevalent in both Finnish and Swedish SD patients.^26^ The variant was absent in the healthy control cell line.

**Fig. 2:**
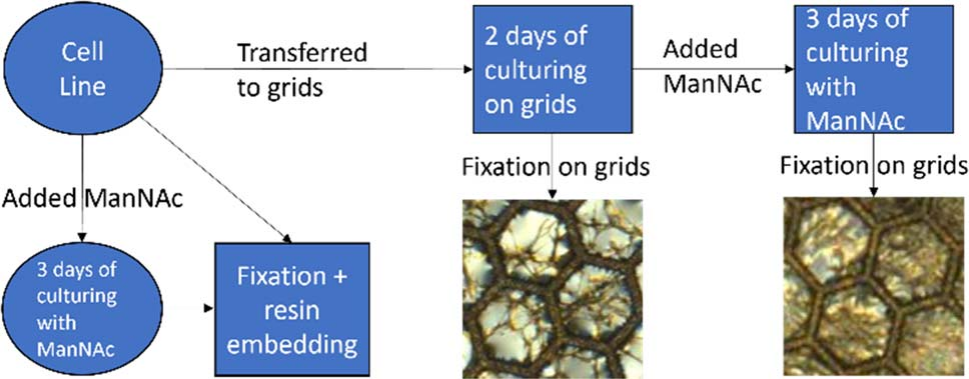
Cell culture workflow for both the SD patient and NHDF control cell lines.

Cell culturing for both cell lines was started at passage nine and they were cultured at the same time through the same cell culture workflow shown in Fig. 2. Each cell line was maintained on 10 cm plates in fibroblast growth medium [Dulbecco's modified eagle's medium (DMEM; Corning, Manassas, VA, USA) supplemented with 10% fetal bovine serum (FBS Good; Pan Biotech, Aidenbach, Bavaria, Germany), 2 mM l-glutamate (Gibco, Thermo Fisher Scientific, Hampton, New Hampshire, USA), and 1% penicillin–streptomycin (GE Healthcare HyClone™, Fisher Scientific)] in a cell incubator at 37 °C and 5% CO_2_. The cells were passaged onto new 10 cm plates to be used in different parts of the experiment. The cells for the resin-embedded samples were grown on new 10 cm plates. For both cell lines, two plates of the cells were cultured in the presence of 30 mM *N*-acetyl-d-mannosamine monohydrate (ManNAc, Thermo Scientific Chemicals) in fibroblast growth medium to increase the amount of sialic acid produced within the cells. After culturing the cells for 3 days and achieving confluency, the cells were fixed and collected at passage 14.

For the non-embedded samples, the cell suspension from the 10 cm plates was plated directly on top of nickel grids (diameter ∼ 3 mm) with Formvar film (polyvinyl formal resin). The film allows the cells to grow on a flat surface between the grid bars to make imaging of the whole cells possible. The grids were placed in 4-well plates for cell culturing. The cells grew confluent on the grids in two days, after which half of the grids were fixed and the other half were moved to new wells with 30 mM ManNAc in fibroblast growth medium. The cells were cultured for three more days in the presence of 30 mM ManNAc after which they were fixed. Fixation of the grid-grown cells happened at passage 15.

### 2.2 Sample preparation

The samples for imaging were either resin-embedded or simply fixed on top of TEM grids. The resin-embedded thin slices were prepared by fixing the cells with 4% paraformaldehyde and 2.5% glutaraldehyde in 0.1 M phosphate-buffered saline for 10 min at room temperature. The cells were then scraped from the bottom of the plates and centrifuged to the bottom of an Eppendorf tube. The pellets were left in the fixative and after drying them with EtOH series they were embedded into the resin material. For STXM imaging, the fixed cells were embedded in a 1 : 1 weight ratio mixture of trimethylolpropane triglycidyl ether and 4,4′-methylenebis(2-methylcyclohexylamine) (TTE : MMHA, Sigma-Aldrich, product numbers #430269 and #369500) without any staining agents. For TEM imaging, the fixed cells were stained with 1% OsO_4_ and uranyl acetate and embedded in EPON resin (glycerol polyglycidyl ether, LX 112, Ladd Research). The embedded samples were sectioned to 150 nm (for STXM) and 70 nm (for TEM) thicknesses using an ultramicrotome (Ultracut, Leica) and mounted on circular copper grids (diameter ∼ 3 mm) with Butvar B-98 (polyvinyl butyral resin, Electron Microscopy Sciences, PA, USA) film.

For the cells grown directly on top of the TEM grids, circular nickel grids (diameter ∼ 3 mm) with Formvar film (polyvinyl formal resin, Electron Microscopy Sciences, PA, USA) were used. The cells were fixed with 4% paraformaldehyde and 2.5% glutaraldehyde in 0.1 M phosphate-buffered saline. Even without the resin embedding, fixation was required to preserve the ultra-fine structures within the cells. The fixed cells were then simply dried with increasing EtOH series. The samples were stored as is without any staining or embedding.

### 2.3 Imaging

TEM imaging was performed with the EPON-embedded samples at Biocenter Oulu using a Tecnai G2 Spirit 120 kV TEM (FEI, Eindhoven, The Netherlands) equipped with a Quemesa CCD camera (Olympus Soft Imaging Solutions GmbH, Münster, Germany). The samples grown on the grid were not imaged with TEM as they were too thick.

STXM-XANES experiments were carried out at the BL4U beamline at UVSOR-III, Okazaki, Japan.^29,30^ Energy scans were performed across the C 1s edge with energy steps of 0.5 eV for 282–284 eV, 0.1 eV for 284–290 eV, 0.2 eV for 290–295 eV, and 1 eV for 295–300 eV ranges. The dwell time was 2 ms. The spatial resolution was limited by the selected step size of 125 nm.

For reference, a C 1s X-ray absorption spectrum of SA, *N*-acetylneuraminic acid (Neu5Ac, 5-(acetylamino)-3,5-dideoxy-d-*glycero*-α-d-galacto-non-2-ulopyranosonic acid) (FUJIFILM Wako Pure Chemical Corporation, Japan) was also measured. The sample was drop-cast on a Si_3_N_4_-membrane.

### 2.4 Data analysis

Data analysis was carried out using aXis2000 software.^31^ Occasional drifting of the images was corrected using alignment procedures implemented in the aXis2000 package. The images were converted to optical densities (ODs) using a reference spectrum *I*_0_ measured from a plain Butvar or Formvar film outside of the thin sections and cells, respectively, or from a Si_3_N_4_ film for the SA reference. Thus, in the presented greyscale STXM images, the lighter the pixel, the more it absorbs at that energy range. For the XANES spectra of the resin-embedded samples, the resin spectrum was subtracted from the cell parts' spectra similar to in previous work (see *e.g.*, ref. 14). For each cell, the subtracted resin spectrum was taken from the same measurement as the cell itself. The subtraction was based on minimizing the shoulder in the spectrum at around 287.2 eV. This was done separately for each sample and each part of the sample. The amount of resin subtracted varied from 55% to 80% depending on the cell part, as the penetration of the resin varied. The subtraction and comparison between the spectra were done using Igor Pro 8.04 (WaveMetrics, Inc. Oregon, USA).

## 3. Results and discussion

### 3.1 Pure sialic acid (Neu5Ac) C 1s X-ray absorption spectrum

**Fig. 3:**
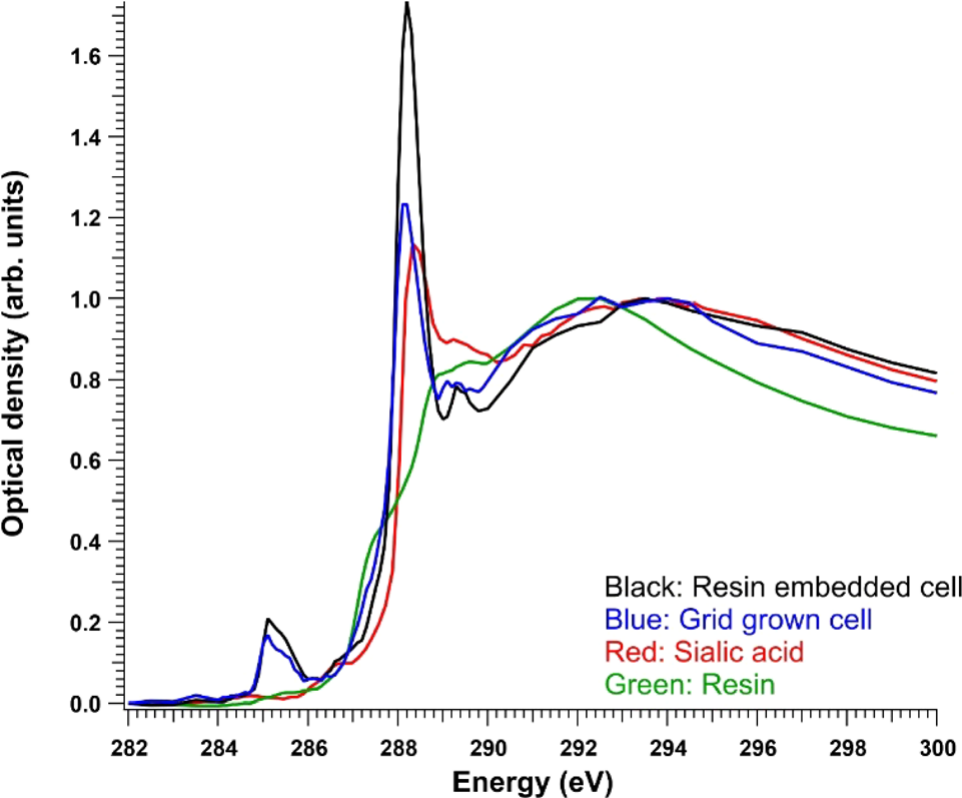
C 1s optical density of the control resin-embedded NHDF cells after resin subtraction (black), grid-grown NHDF cells (blue), pure sialic acid (red), and TTE : MMHA resin (green) normalized to the post-edge maximum.

To facilitate the following discussion, the reference spectrum of pure sialic acid and the average absorption signals from cells are presented first. Fig. 3 shows a comparison of the C 1s XANES (in optical density) of Neu5Ac and the average signal from the whole resin-embedded cells after resin subtraction and a region of a cell grown on a grid. A TTE : MMHA resin spectrum is also shown for reference. Neu5Ac consists of nine-carbon sugar neuraminic acid, in which an acetyl group is attached to the amino group. Thus, it also has an amide group, but its C=O C 1s → π* transition was at a slightly higher energy (∼288.3 eV) compared to the observed cell spectra. After the C=O peak, there was a broad “post-peak” feature at around 289.5 eV. In similar amino sugars, transitions around this energy have previously been tentatively assigned to originate from C 1s → 3p/σ* transitions in the C–OH groups of the sugar.^32^ The maximum of the Neu5Ac post-edge feature was at around 294 eV, like in the cell spectra.

### 3.2 Cells

For both sample preparation types (resin-embedded and cells grown on grids), four different sample sets were studied: NHDF cell line without the addition of ManNAc (called NHDF), with ManNAc (called NHDF + ManNAc), SD cell line without ManNAc (called SD), and SD cell line with ManNAc (called SD + ManNAc). The NHDF cell line served as a control exhibiting the normal function of sialin. Example spectra for the NHDF control cell lines are presented in Fig. 3. The cell spectra showed typical transitions for the protein-rich specimen: at around 285 eV, an asymmetric peak from C 1s → π* transitions in unsaturated hydrocarbons and aromatic substances, and at 288.2 eV, the C=O C 1s → π* transitions in amides, similarly to *e.g.*, the albumin spectrum.^19,33^ There was a small peak, especially visible in the resin-embedded sample, at 289.3 eV, which has previously been assigned as C=N C 1s → π* transitions.^34^ At the same energy, there is a C–OH bond-related transition in sugars.^32,35^ In the SD cell line, sialic acid is expected to accumulate in lysosomes. In previously reported patient cases with free SA storage diseases, electron microscopy of skin biopsies and cultured fibroblasts has revealed cytoplasmic vacuoles and enlarged lysosomes.^25,36^ In our previous study using *Slc17a5* knock-out mice, we observed vacuoles in liver and kidney tissues.^22^ Here, the expectation was to observe vacuoles in fibroblasts as well, and aided by the spectroscopic signature of free sialic acid, shown in Fig. 3, compare its spatial distribution in the cells. While resin embedding is known to preserve the structure of cells, the non-embedded, grid-grown cells were studied to obtain “purer” spectroscopic signatures that would not be masked by resin. In the following sections, the resin-embedded samples and grid-grown samples are discussed separately.

#### 3.2.1 Resin-embedded samples

**Fig. 4:**
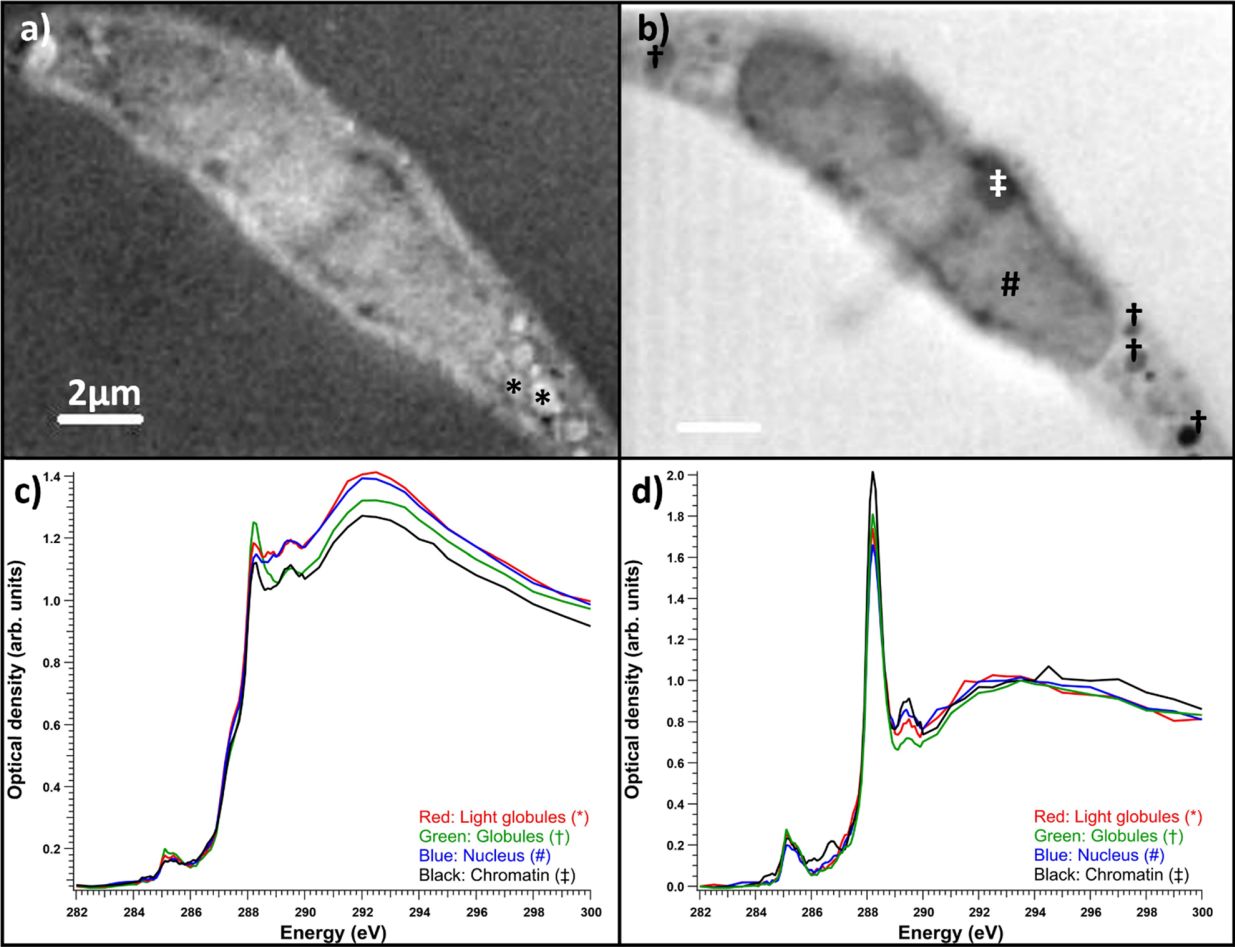
Salla disease fibroblast with accumulation material. (a) Optical density image averaged over the energy range of 287.50–288.50 eV, (b) optical density image averaged over the energy range of 288.60–300 eV. (c) Raw spectra of the optical densities averages of areas: red is for the light globules marked with *, green is for the dark globules marked with †, blue is the inside of the nucleus marked with #, and black is the chromatin marked with ‡. (c) Spectra before and (d) after resin subtraction, normalized to the post-edge maximum.

The benefit of resin embedding is that it preserves the ultrastructural features, mitigates the effects of radiation damage, and provides a constant thickness throughout the samples, and thus, the cells in resin-embedded samples can be easily measured. Here, the TEM analysis showed an increase in empty vacuoles and multilamellar structures in the SD cell line compared to the NHDF control line; however, this was not as drastic as reported in the literature (see ESI Fig. S1† for TEM images). Despite being from a confirmed SD patient cell line, most of the cells had no structures corresponding to lysosomal accumulation. In the cells that had vacuoles, in most cases the spectrum extracted from the vacuoles corresponded to pure resin spectrum. However, in some cases, like close to the fibroblast nucleus shown in Fig. 4, there were vacuolar structures or globules with distinct spectra. Fig. 4a shows an OD image of the fibroblast in the energy range 287.50–288.50 eV, *i.e.*, averaged over the main peak of the cell spectra. Here, all the globules (marked with stars (*) in Fig. 4a) and daggers (†) in Fig. 4b)) are light-colored, absorbing almost equally. However, in the post-edge range shown in Fig. 4b), the globules marked with dagger clearly absorbed less than surrounding cell material, and much less than the embedding resin. The XANES spectrum before the resin signal subtraction of these globules (Fig. 4c), green line) differed from other selected cell parts' spectra. The absorption was the strongest around 288.2 eV, similar to proteins, and the main peak seemed to display broadening toward higher energies. However, when the resin contribution was subtracted (Fig. 4d)), it became evident that most of the differences in the absorption contrast were due to the differences in resin penetration in to the cell parts, as all the globules as well as the region inside the nucleus showed similar protein-like spectra. The variation in relative intensities of the main absorption peak and smaller features could be due to material or protein concentration differences in these structures. Resin subtraction is a delicate process. In our case, the subtraction was performed by minimizing the shoulder peak caused by the resin at around 287.2 eV. As the slope of the spectrum is steep at this energy, even slight changes in the resin subtraction can change the relative intensities of the peaks in the sample. This combined with the differences in resin penetration in to different parts of the cell means that each spectrum has to be processed separately before calculating any averages. After resin subtraction, the chromatin spectrum (Fig. 4d, black line) extracted from a region indicated with a double dagger (‡) in Fig. 4b) clearly differed from the rest of the sample. Chromatin has small extra peaks around 286, 286.7, and 287.4 eV. These energies matched the peaks of DNA measured by Shinohara *et al.*^19–21^ Our measurement of chromatin was in line with their results for DNA and histone, which are the major components of chromatin. A faint line in the middle of the cell in Fig. 4b) was due to radiation damage caused by a focus scan performed right before the STXM-XANES acquisition was started. See ESI† for a discussion about the radiation damage. Usually, the areas of interest are avoided when doing focus scans.

**Fig. 5:**
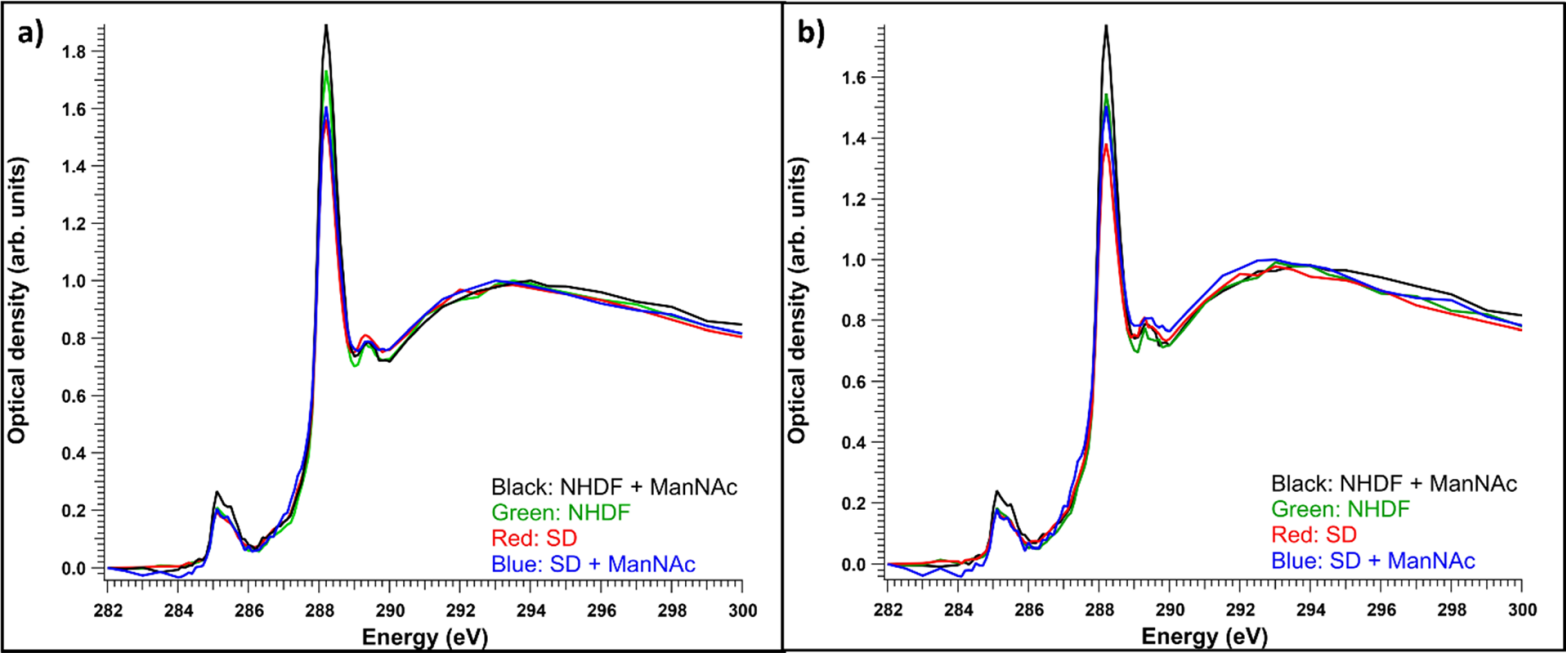
(a) Average optical densities of all measured cell samples for each sample type, (b) average optical densities of cytosols from all the measured cells for each sample type, (*n* = 4–6 for each sample type), normalized to the post-edge maximum.

Like observed before,^22^ even if the vacuolic structures would have originally contained accumulating material, it likely would not have survived the sample processing. Thus, it turned out to be useful to compare the total and cytosolic spectra of different sample types. The average absorption spectra (optical density) of the whole measured regions for each sample type are presented in Fig. 5a. Each of the spectra are averages of 4–6 measured cells. The resin contributions have been subtracted from the spectra and they have been normalized to the post-edge maximum to allow comparing the relative intensity ratios. The NHDF cells cultured with ManNAc had the highest absorption at around 288.2 eV, while the SD patient cells cultured with or without ManNAc had the lowest absorption at that energy. The overall similarity in the shape of the spectra (typical protein-like spectrum, as discussed in Section 3.1) indicated the difference between the samples was in their protein content and concentration and could not be explained by the presence of SA in its free form, as there was no clear broadening of the main absorption feature at 288.2 eV. Also, the relative intensities of the 288.2 eV peak and 295 eV peak remained similar, but the presence of SA would have changed this ratio as it does not have transitions around 285 eV. The measured regions were not always from the same parts of the cell. In some cases, the nucleus was a large part of the region while sometimes there was no nucleus present. To consider structural differences, spectral analysis from regions representing the cytosol was also performed. The average cytosol spectra for each sample type are presented in Fig. 5b). The results are remarkably similar to the whole cell spectra presented in Fig. 5a), with NHDF + ManNAc having the highest relative protein-like signal and SD the lowest. In NHDF cells, ManNAc increases the absorption of the pre-edge peaks in both the cytosol and entire cell, while in the SD samples the intensity increase was only observable in the cytosol. The lower protein content in the cytosol of the SD cell lines compared to NHDF cell lines can indicate that the glycoproteins to which SA is bound in the cytosol are affected by the disease.

#### 3.2.2 Fibroblasts grown directly on grids

To remove processing steps that might affect the chemical structure in the samples, cells were grown directly on top of the TEM grids. Fixing the cell on top of the grids removes the need to subtract the resin spectrum in data processing, making it easier, but on the other hand it removes the possibility to cut them uniformly thin enough for STXM imaging. To grow the fibroblast on top of the grids, Formvar-coated nickel grids instead of Butvar-coated copper grids were used, as the cytotoxicity of the copper would kill all the cells within the culture well. With nickel grids, there did not seem to be any more dead cells compared to cells grown on a 10 cm plate. An example of how fibroblasts grew on top of a grid is presented in Fig. 1. For each sample there were three parallel sample grids in the same well. There were noticeable differences in cell confluency within these parallel samples but no clear trend in cell confluence when comparing the patient and control samples. The addition of ManNAc into the culture medium did not seem to affect the cells negatively and the three extra days of cell culture caused the fibroblasts to grow overconfluent, as shown in Fig. 1.

**Fig. 6:**
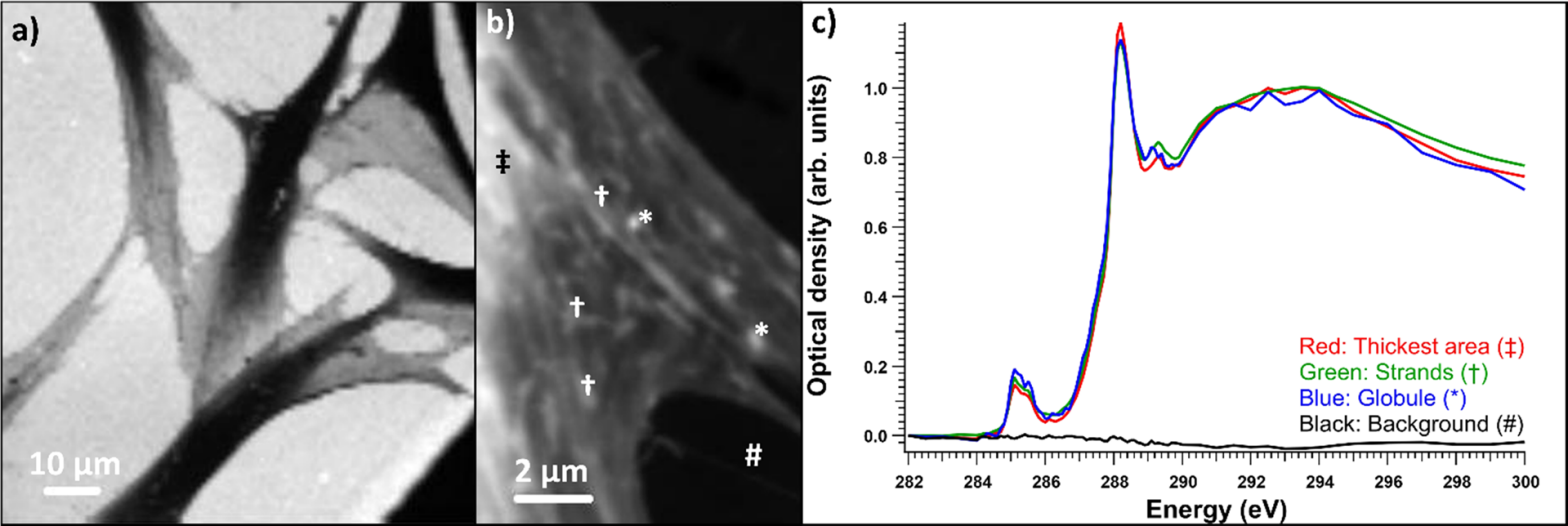
SD fibroblasts grown on grids. (a) Single energy scan of transmission through fibroblasts measured at 292.5 eV, (b) close-up optical density energy stack of the fibroblast edge area, (c) normalized average spectra; red is the thickest area marker with ‡, green is for the strand like structures marked with †, blue is for the globular structures marked with *, and black is the background marked with #.

**Fig. 7:**
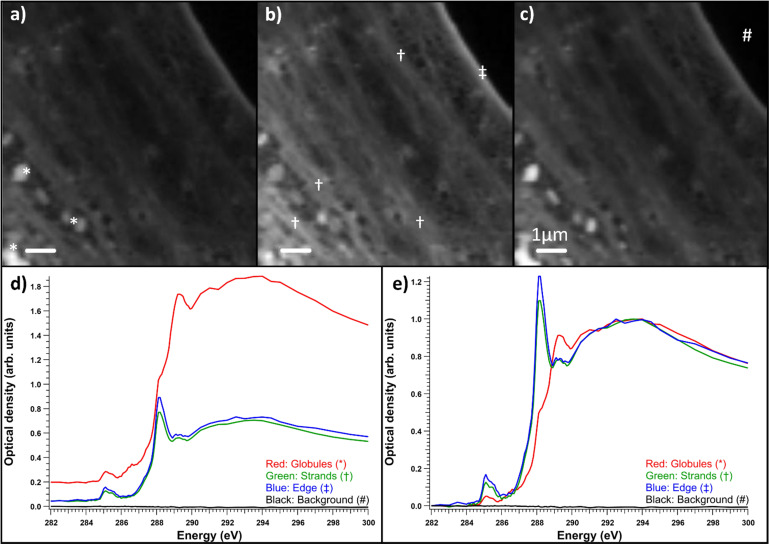
Optical densities of control NHDF fibroblasts with globules in them. (a) Energy range of 282–287.5 eV, (b) energy range around the protein peak at 287.6–288.5 eV, (c) energy range of 288.6–300 eV after the protein peak. The spectra are the optical density averages of the marked areas of interest. Red is for the globules marked with *, green is for the strands that present a strong protein-like spectrum marked with †, blue is the edge of the cell marked with ‡, and black is the background marked with #. (d) Average measured optical densities and (e) the same spectra normalized by the post-edge maximum.

Fibroblasts grown on nickel grids were too thick to obtain a measurable signal through the thicker parts of the cell, *e.g.*, the nucleus (Fig. 6a). In the energy range of the C 1s edge, the attenuation length in dried cells can be below 0.3 μm,^37^ while the nuclei of whole two-dimensionally grown fibroblasts can be a few micrometers thick.^38^ However, transmission was high enough in the fringes of the fibroblasts and the measured areas seemed to consist mostly of cytoskeletal structures, as shown in Fig. 6b. Most the differences seemed to be thickness related as the samples were not uniformly thick, unlike the thin embedded sections. Fig. 6c) shows the normalized optical densities from different parts of the SD fibroblast. Again, all the areas showed the typical absorption spectrum of proteins with clear peaks at ∼285 and 288.2 eV. There was some variation in the energy of the third peak around 289.3 eV and over the ionization threshold between the regions. The general shape of the spectrum remained the same for each area but there were some differences between absorption strength at different energies. This was the case for most samples and there were no significant differences between sample types (SD, SD + ManNAc, NHDF, NHDF + ManNAc). This indicates that the cells were mostly left with their protein structure intact after EtOH drying. Other cytoplasmic content was not detectable. This is in line with the results from the thin slices after resin subtraction. Thus, even if the cells grown directly on grids avoid a major sample modification step (resin embedding), the EtOH drying step required to preserve the structure possibly alters the chemistry. Unlike with thin slices, comparisons between the OD averages of the differently cultured cells were not made. This was due to the fact that the thickness was the major difference between the samples and varied massively within and between each sample. In some NHDF fibroblasts, strongly absorbing globules were observed, as shown in Fig. 7. The detected areas (marked with * in Fig. 7a)) with differing spectra were thicker than the surrounding area as seen in Fig. 7d) but still thin enough to have transmission. The absorption in these areas was the strongest around 289.3 eV and the spectrum resembles the spectra of sugars without amino groups.^32,35^ Most likely, these were deposits of sugars either in the fibroblasts or remnants from the cell culture medium outside the cell. The regions had a similar amount of absorption at 288 eV as the rest of the cell, indicating that the sugar signal was superimposed on the protein-rich cell structure (Fig. 7d)). Fig. 7e) shows a comparison of the spectra after normalization to the post-edge maximum.

## 4. Conclusions

NHDF and SD patient-derived cells were successfully imaged using STXM-XANES. The patient fibroblasts did not exhibit as strong a phenotype as previously reported in the literature. Most of the vacuoles had a bare resin spectrum and most the spectra from the cell structures resembled that of a protein. It seems that the other materials within the cells were lost during the sample preparation process, or their relative amounts were too small for STXM-XANES to resolve them from proteins. However, structural proteins could be measured, and chromatin had its own distinct spectrum, which could be seen after subtracting the resin spectrum. Compared to literature, this measured spectrum was consistent with chromatin being made of DNA and histone. This indicates that STXM-XANES can be used to measure the structural components of cells even when embedded in resin material, but more soluble materials are lost when samples are prepared this way. Especially, while there was a clear difference in the relative protein concentration between the two cell lines, there was no increase in SA spectrum in the vacuoles. The SD cell line had a reduced relative protein concentration compared to the NHDF cell line. The addition of ManNAc increased the relative protein concentration for both the NHDF and SD cell lines in cytosol, but only for NHDF when measuring the whole cell area.

The cells were also imaged without resin to reduce the amount of processing of the samples. The removal of resin from the equation by using whole grid-grown fibroblasts instead of resin-embedded thin samples provided well-resolved spectra, but free SA remained undetected. The thickest parts of grid-grown fibroblasts were several micrometers thick and thus too thick for soft X-ray imaging, but the edge areas of the cells could be measured. Even without the resin embedding, most of the areas had protein spectral signals consistent with the spectra from thin sections after resin subtraction. Thus, it could be concluded that the resin embedding was not the main problem but the drying through EtOH series to preserve the structure of the cells may dissolve accumulating free molecules. The grid-grown cells had globular structures with a substantial increase in the relative absorption at around 289.3 eV, and based on their XANES spectra they were identified to contain non-amino sugars. While these regions were exceptions and not the rule, they give a promise of the future use of STXM-XANES for the chemically resolved imaging of cells without staining agents. All the samples were fixed with glutaraldehyde and paraformaldehyde, which introduces some chemical changes *via* crosslinking.^39^ Cryo-preservation can avoid this sample preparation step, and cryogenic sample environments are more commonly becoming available at soft X-ray STXM beamlines, offering interesting possibilities for sample spectromicroscopic imaging close to their native state.^30,40,41^ However, the thickness of cryo-preserved cells and soft tissue samples still poses a challenge for transmission spectromicroscopic imaging at the C 1s edge, but developments in workflows for cryogenic TEM^42^ can also benefit this field.

## Data availability

The STXM data for this article are available at Fairdata.fi-service: https://doi.org/10.23729/d441d0e0-7ce7-4f76-9c1a-ef4ccaee54ff. The TEM data supporting this article have been included as part of the ESI.†

## Author contributions

Tuomas Mansikkala: writing – original draft, investigation, formal analysis, visualization, funding acquisition. Salla M Kangas: writing – review & editing, methodology, conceptualization, supervision. Ilkka Miinalainen: writing – review & editing, methodology, conceptualization, supervision. Pia Angervaniva: investigation. Niklas Darin: resources, writing – review & editing. Maria Blomqvist: resources, writing – review & editing. Reetta Hinttala: writing – review & editing, funding acquisition. Marko Huttula: writing – review & editing, funding acquisition. Johanna Uusimaa: writing – review & editing, resources, conceptualization, funding acquisition. Minna Patanen: writing – review & editing, investigation, validation, conceptualization, funding acquisition, supervision.

## Conflicts of interest

There are no conflicts to declare.

## Supplementary Material

RA-014-D4RA05520A-s001
