# Supplementary material for: Soft X-ray spectromicroscopy of human fibroblasts with impaired sialin function
Source: RSC Adv. 2024 Sep 10;14(39):28797–806. doi: 10.1039/d4ra05520a (PMC11385984; doi:10.1039/d4ra05520a)
Supplement: RA-014-D4RA05520A-s001 [file RA-014-D4RA05520A-s001.pdf]

Supplementary Information (SI) for RSC Advances.
This journal is © The Royal Society of Chemistry 2024

Electronic supplementary information for:

Soft X-ray spectromicroscopy of human fibroblasts with impaired sialin function

Tuomas Mansikkala, Salla M Kangas, Ilkka Miinalainen, Pia Angervaniva, Niklas Darin, Maria Blomqvist, Reetta Hinttala, Marko Huttula, Johanna Uusimaa, Minna Patanen

Transmission electron microscopy images

Transmission electron microscopy images of cultured Salla disease (SD) patient and NHDF control cell lines are presented in Fig. S1. In both cases we saw empty vacuolar structures but in SD cell line they were more frequent and numerous.

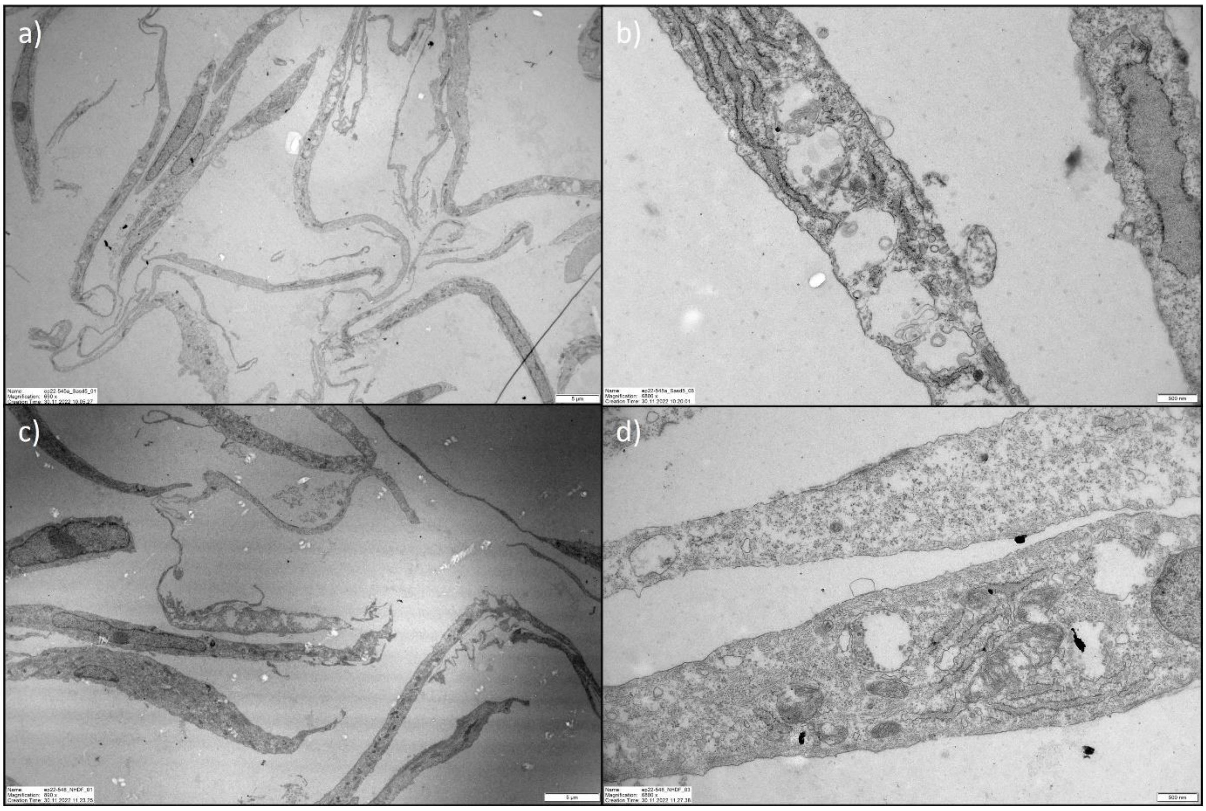

Figure S1. Representative TEM images of the measured cell lines a) is the overview of SD cell line and b) is close up image of SD cell line. c) is the overview of NHDF control cell line and d) is close up image of NHDF cell line. Scale bar in 5 µm for overview images (a & c) and 500 nm for close ups (b & d).

Radiation damage

Effects of radiation damage from the focus scans of the X-ray beam are presented in Fig. S2. The C=C C 1s → π* spectral feature at 285 eV gains intensity and a new feature at around 286.5 eV appears, while the intensity of the absorption edge decreases, in agreement with study of Wang *et al*. on PMMA polymer [1]. This is interpreted as CO_2 loss from the sample. In Fig. S2, the OD image is made as an average image of energies at 285 eV, highlighting the

high C=C intensity areas. The thin white line is formed during a focus scan, where the same line is scanned at single energy over and over again while changing the distance between the sample and the Fresnel zone plate. Thus, the radiation damage there is much more severe (also the dwell time is typically 5 ms in the focus scans) than in the actual imaging measurement but demonstrates the spectral changes clearly. The effects are more pronounced in the pure resin part compared to inside of the cell containing less resin.

The spectral changes can be different in non-embedded cells. Wang *et al*. also showed that in protein (fibrinogen) sample, there is no increase in intensity at 285 eV in contrast to PMMA, and but there is a loss of the C=O C 1s → π* signal [1]. In this case, decarboxylation is not likely to be an important radiation damage route, but Wang *et al*. suggest C=N double bond formation and elimination of water to be more likely.

We did not systematically make a radiation damage test for the native cells. Despite small deformation (drifting or shrinkage) of the samples during the energy stack acquisition, we did not observe other alterations under the beam. The spectra from thin and thicker parts of the grid-grown cells are similar to resin-subtracted spectra of embedded samples, which makes us believe that during the scan, the samples are not significantly damaged. If the damage occurs during the scan, it is likely to affect mostly the relative intensity of the post edge compared to the pre-edge, which is used more for the chemical speciation. Most of the damage is caused at the energies in the post edge region due to its strong absorption and this damage would become important if the same area would have been imaged again.

References

[1] J. Wang, C. Morin, L. Li, A.P. Hitchcock, A. Scholl, A. Doran, Radiation damage in soft X-ray microscopy, J. Electron Spectrosc. Rel. Phen. 170, 25-36 (2009) https://doi.org/10.1016/j.elspec.2008.01.002

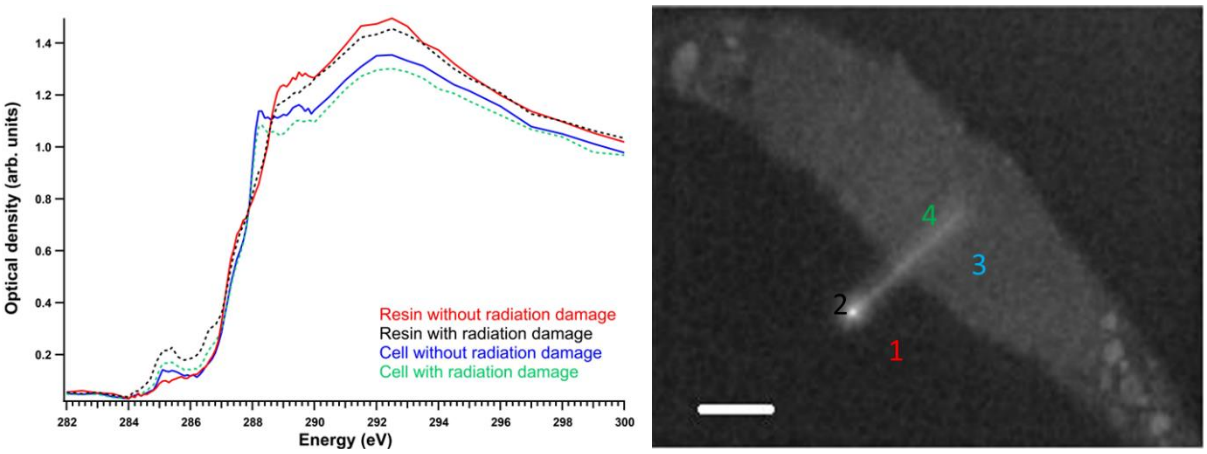

Figure S2. Changes in spectra caused by radiation damage from the focus scan of the X-ray beam. Red (1) and blue (3) are from areas near the focus scan damage. Dashed lines are spectra from the focus scan area. Black (2) is from outside the cell and green (4) is from within the cell.
